# Correlation between *CFTR* variants and outcomes of ART in patients with CAVD in Central China

**DOI:** 10.1038/s41598-022-26384-8

**Published:** 2023-01-05

**Authors:** Xiaowei Qu, Lingyi Li, Chenchen Cui, Ke Feng, Yanqing Xia, Feng Wan, Cuilian Zhang, Haibin Guo

**Affiliations:** Henan Provincial People’s Hospital, Henan Provincial Reproductive Hospital, People’s Hospital of Henan University, Zhengzhou, 450003 Henan China

**Keywords:** Biological techniques, Genetics, Medical research, Urology

## Abstract

Biallelic variants in Cystic Fibrosis Transmembrane Conductance Regulator (*CFTR*) are the main pathogenic factor of congenital absence of the vas deferens (CAVD), including congenital bilateral absence of the vas deferens (CBAVD) and congenital unilateral absence of the vas deferens (CUAVD). However, there are few reports about the correlation between *CFTR* variant and outcomes of assisted reproductive technology (ART) in CAVD patients of China. In this study, 104 patients with CAVD were recruited in Central China, and provided gene detection by the whole-exome sequencing, among them 69% (72/104) carried at least one variant in *CFTR* and one carried adhesion G protein-coupled receptor G2 (A*DGRG2)* variant. A total of 81 CAVD patients were treated with ART, of which 21 and 60 carried none or at least one variant in *CFTR*, respectively. The fertilization rate, cleavage rate, effective embryo rate, implantation rate, clinical pregnancy rate and live birth rate per fresh embryo transfer were compared between patients with and without *CFTR* variants. It was found that the ART outcomes had no significant difference whether the patients carried the *CFTR* variant or not. In addition, all of the offspring were healthy after follow-up. In conclusion, rare *CFTR* variants may play a major role in patients with CAVD in Central China, which were greatly different from other descent. There was no significant difference in ART outcomes in CAVD patients with or without *CFTR* variants. The limitations of this study were that there was no statistical analysis of the sperm quality through TESA and conclusions were relatively limited due to the small sample size of the study.

## Introduction

Congenital bilateral absence of the vas deferens (CBAVD) accounts for about 2–6% of male infertility and 25% of obstructive azoospermia (OA), which is a significantly contributing factor to male infertility^1^.

At present, it is mainly caused by the gene variant of Cystic Fibrosis Transmembrane Conductance Regulator (*CFTR*)^2^. CFTR is a chloride channel protein and is widely expressed in the respiratory system, digestive system, reproductive system, endocrine system and sweat glands, which maintains the balance and homeostasis of electrolyte in human body. Loss of CFTR protein might cause different clinical phenotypes in patients, including cystic fibrosis (CF), diffuse bronchiectasis, acute or recurrent pancreatitis and CAVD. When the level of CFTR protein drops to about 10% of the normal value, CF or CFTR-Related disorders might occur. Only a slight decrease in CFTR protein expression can lead to CBAVD. Therefore, CBAVD is considered to be a mild clinical manifestation of CF and is defined as a CFTR-Related disorders^3^. *CFTR* variant can cause dysfunction of chloride ion channel in cell membrane, making cells unable to regulate the flow of chloride ion and water molecules, thus leading to the exclusion of viscous secretion produced by reproductive tract, which will lead to vas deferens obstruction and degeneration during embryo development, resulting in subsequent development of CBAVD^4,5^. *CFTR* variants occur in up to 40% of non-vasectomized men with OA^6^. In a meta-analysis study, 78% of men with CBAVD carried one or two (a severe and a mild or two mild) *CFTR* variants^7^. CBAVD was found in ~ 44% of a relatively unselected population of azoospermic men with p.Phe508del/ p.Arg117His variants^8^. The variant frequency and hot spots of *CFTR* gene in Caucasians were demonstrated to be significantly higher than that in individuals of Asian descent. Two different variants in *CFTR* gene (compound heterozygote) can be found in 63% to 83% CBAVD patients^3,9,10^. Moreover, a few CBAVD patients are caused by variants in the X-linked *ADGRG2* (adhesion G protein-coupled receptor G2) gene^11^. To rule out X-linked transmission of CBAVD, *ADGRG2* gene testing should be performed in *CFTR* negative patients^12^.

There was no significant abnormality in testicular sperm in patients with obstructive azoospermia, and most CAVD couples can have a biological child through assisted reproductive technology (ART). Therefore, the genetic etiology is often ignored, and then the research on assisted reproductive outcomes and offspring birth defects are often ignored.

It has been reported that genetic variants in CBAVD patients can affect spermatogenic function and sperm quality, thereby interfering with the ART outcome. In addition, there is a risk of passing on pathogenic genetic variants to offspring^13^. A novel comparison of *CFTR* variants and fertility outcomes of patients with either CF or CBAVD alone found that CF men were more likely to exhibit lower sperm quality, greater difficulty with sperm retrieval, and worse ICSI outcomes compared with CBAVD-only patients^14^. Another study showed that no differences were found when comparing presence of severe CF, common *CFTR* gene variants and ICSI-related parameters^15^.

Although discordant data had been reported concerning the correlation between *CFTR* variants and outcomes of ART in patients with CAVD, considering that CF is an uncommon and life-threatening disorder, *CFTR* gene testing and counseling is strongly recommended for patients and their partners^4^.

However, there are not enough reports on *CFTR* gene screening in China, and the distribution of *CFTR* variants remains unclear. In addition, there are few studies on assisted pregnancy outcomes in patients with *CFTR* variants.

In this study, 104 Chinese CAVD patients without any typical CF symptoms were included. *CFTR* gene was screened by Whole-exome sequencing. Among them, 81 patients were treated with Intracytoplasmic sperm injection-embryo transfer/Testicular sperm aspiration (ICSI-ET/TESA). The results may provide a reference for clinical diagnosis, genetic counseling and assisted reproduction of Chinese patients with CAVD.

## Material and methods

### Study population

**Figure 1:**
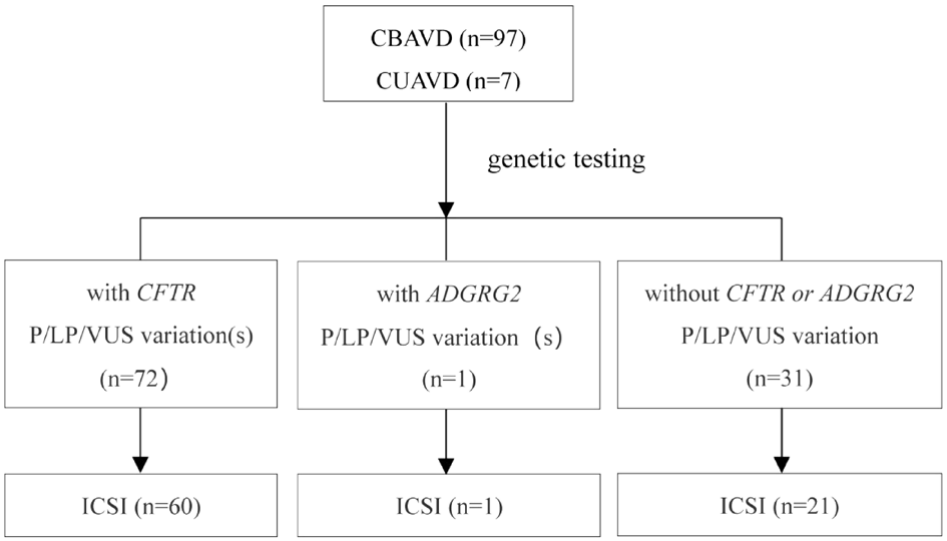
Study flow chart. *CBAVD* congenital bilateral absence of the vas deferens, *CUAVD* congenital unilateral absence of the vas deferens, *ICSI* intra cytoplasmic sperm injection.

From May 2018 to Apr 2022, 104 patients with CAVD (CUAVD or CBAVD) from Central China were enrolled (Fig. 1). Computer assisted semen analysis (CASA), testicular volume and sex hormone level were detected, and scrotal color Doppler ultrasonography was performed. No patient showed typical CF symptoms. Blood samples were collected from patients with CAVD and their parents or spouses for *CFTR* screening. Among them, 81 patients were treated with ICSI-ET/TESA. This research was approved by the Medical Ethics Committee of Henan Provincial People's Hospital, and the guidelines outlined in the Declaration of Helsinki were followed. All participants signed the written informed consent form.

### Whole-exome sequencing and validation

Genomic DNA was extracted from blood samples using the DNeasy Blood & Tissue Kit (Qiagen). Whole-exome sequencing of samples were prepared using IDT xGen Exome Research Panel V1.0 (Integrated DNA Technologies). The quantity of sequencing library was assessed by Qubit 2.0 fluorometer (Thermo Fisher Scientific). The quality and size of libraries were measured by 2100 Bioanalyzer High Sensitivity DNA Assay (Agilent Technologies).

For next-generation sequencing, the qualified libraries were applied to 2 × 150-bp paired-end sequencing on the Illumina NovaSeq platform (Illumina, San Diego, USA). FASTQ files were aligned to the human reference genome (hg19/GRCh37) by BWA v0.7.13^16^. Variants (single nucleotide variants and indels) were genotyped from recalibrated BAM files by GATK 4.0 and annotated using ANNOVAR against multiple databases, including HGVS variant description, population frequency, disease or phenotype and variant functional prediction. Variants were classified as pathogenic, likely pathogenic, variant of unknown significance (VUS), likely benign, or benign following the American College of Medical Genetics (ACMG) guidelines^17–19^. Copy number variants were called by DNA copy R package, filtered and classified by ACMG guidelines and manually checked using the Integrative Genomics Viewer^20–22^. Confirmation of variant and the familial co-segregation analysis were performed by Sanger sequencing.

### Surgical sperm extraction

Sperms of 81 patients with CAVD were obtained by TESA. After local anesthesia, they were punctured with a disposable syringe, and a small amount of testicular tissue was aspirated. After grind pre-treatment procedure, the number and morphology of sperm were observed under an optical microscope.

### ICSI assisted pregnancy

In this work, the spouses of 81 patients with CAVD received controlled ovulation hyperstimulation. The daily dose of FSH injection was adjusted according to women's Oocytes, ovarian reserve and various responses to ovarian stimulation. Monitoring of follicular development with ultrasonography scanning and follicular aspiration was performed at least 36 h after HCG trigger. Sperms with relatively normal morphology were selected under a 400-fold microscope, and then ICSI was performed. In addition, on the third day of embryo transfer, all transferred embryos were at least 6 cells with blastomeres were uniform, and fragmentations were < 20%. Serum β-HCG was detected 14 days after transplantation, when β-HCG ≥ 50U/L, luteal support treatment was maintained. It was a clinical pregnancy until 4 weeks after transplantation when the gestational sac was found by transvaginal ultrasound. ICSI-ET results included fertilization rate, available embryos, implantation rate, clinical pregnancy rate, abortion rate and live birth rate.

### Statistical analysis

Data analyses were performed using Statistical Package for Social Sciences version 24.0 (SPSS IBM Corporation, Armonk, NY, USA). Data were compared using the Wilcoxon rank sum test, t-test or chi-squared test, where appropriate. Statistical significance was defined as a two-tailed p-value of 0.05.

### Ethical approval

This research was approved by the Medical Ethics Committee of Henan Provincial People's Hospital, and the guidelines outlined in the Declaration of Helsinki were followed. All participants signed the written informed consent form.

## Results

### Clinical characteristics of subjects

In 104 patients with CAVD diagnosed by ultrasonography and physical examination, five of them were observed with absence of bilateral seminal vesicles, one of them were observed with atrophy of bilateral seminal vesicles, and no typical clinical phenotype of CF was observed. Chromosomal karyotype analysis and sex hormone examination showed that all of them had normal 46, XY, FSH, LH, and TT. Furthermore, no sperm was observed in their semen after centrifugation.

### Genetic variant screening

**Figure 2:**
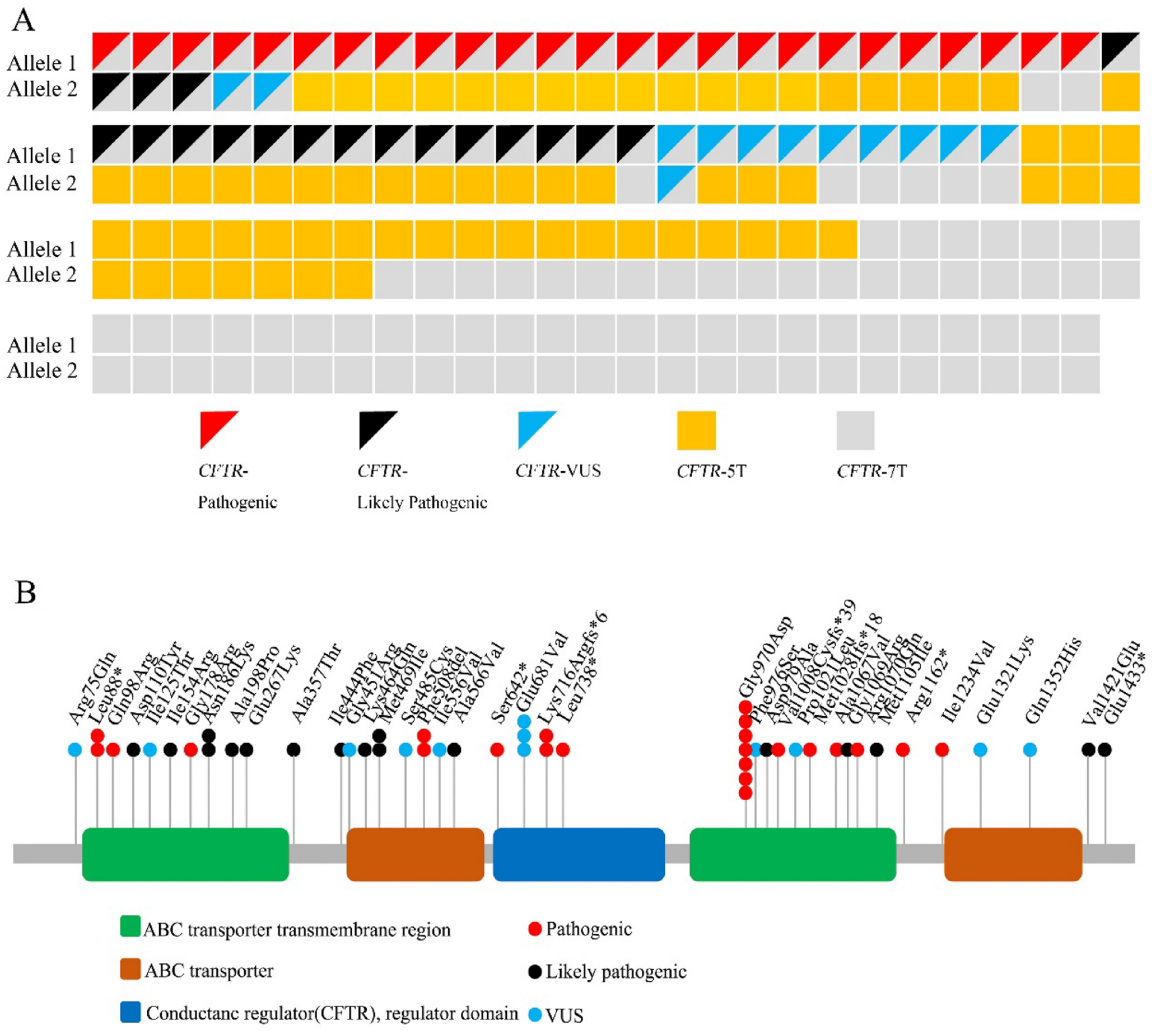
*CFTR* gene variants. (**A**) Pathogenic variants, Likely Pathogenic variants and variants of uncertain significance (VUS) of *CFTR* are shown for the 103 CAVD patients. (**B**) The *CFTR* variants leading to amino acid changes are marked onto protein domains.

69% (72/104) of CAVD patients carried at least one *CFTR* variant, and 44 reportable *CFTR* variants were identified in 72 CAVD patients. Of these, 16 variants were classified as pathogenic, 17 variants were classified as likely pathogenic, and 11 variants were recognized as variants of uncertain significance. In addition, we found one *ADGRG2* variant (c.2041A > G, p.Met681Val) that was classified as likely pathogenic (Figs. 1, 2 and Suppl. Table 1). There were no hot spot variants or associated variants in other genes such as *PANK2, SLC9A3, SCNN1B* and *CA12.* No pathogenic or likely pathogenic *CFTR* variants were found in spouses of CAVD patients carrying *CFTR* variants.

### Population frequency of IVS9-5 T

In all 103 CAVD patients without *ADGRG2* gene variant, 46 (44.7%) carried 7 T/7 T alleles, 47 (45.6%) carried 5 T/7 T alleles, and 10 (9.7%) carried 5 T/5 T alleles. No CAVD patients carried the 9 T allele (Fig. 2 and Suppl. Table 1).

### Sperm recovery results

81 patients with CAVD obtained sperms by TESA. Through microscopic observation, there were no serious head deformity sperms, and occasionally motile sperms could be seen.

### Reproductive outcomes of ICSI-ET/TESA

In this study, 81 patients with CAVD (60 patients with *CFTR* gene variants and 21 patients without *CFTR* or *ADGRG2* gene variant) were treated with ICSI-ET/TESA (Fig. 1). As showed in Table 1, there were no significant differences in age, and the number of MII oocytes retrieved between the two groups. We found that there were no significant differences in fertilization rate, cleavage rate, available embryo rate, implantation rate, clinical pregnancy rate and live birth rate per fresh embryo transfer (ET) whether they carried *CFTR* gene variant or not. In addition, all the offspring were healthy and without typical CF symptoms after follow-up.

**Table 1:** Baseline, embryological characteristics, and reproductive outcomes of ICSI-ET/TESA in patients with CAVD.

Characteristic	*CFTR* variant	No *CFTR* variant	*P* value (t-test)	*P* value (Wilcoxon rank sum test)
n	60	21		
Male age (years)	28.3 ± 3.8	28.4 ± 4.7	0.9	0.7
Female age (years)	27.9 ± 3.9	27.8 ± 4.1	0.9	0.7
MII oocytes (n)	10.3 ± 4.7	10.0 ± 6.4	0.8	0.4

Characteristic	*CFTR* variant	No *CFTR* variant	*P* value (chi-squared test)	
Fertilization rate	75.0% (461/615)	71.3% (149/209)	0.3	
Cleavage rate	96.3% (444/461)	97.3% (145/149)	0.7	
Available embryo rate	55.9% (248/444)	60.7% (88/145)	0.4	
Implantation rate/fresh ET	63.3% (38/60)	56.3% (9/16)	0.8	
Clinical pregnancy rate/fresh ET (n)	73.7% (28/38)	70.0% (7/10)	1.0	
Live birth rate/fresh ET (n)	47.4% (18/38)	40.0% (4/10)	1.0	

*MII oocytes* metaphase II oocytes.

## Discussion

The diversity of clinical phenotypes of CF is related to the amount of protein synthesis and/or activity due to *CFTR* gene variants as well as to the rate/the level of CFTR protein in different tissues. Vas deferens is susceptible to *CFTR* gene variant, and even a low level of *CFTR* transcripts in bronchial epithelial cells is sufficient for the maintenance of normal airway function, which might be one of the reasons for the absence of other clinical manifestations in CAVD patients with *CFTR* gene variants^23,24^.

Researchers have carried out extensive variant screening in exons of the *CFTR* gene and exon–intron junctions (splice sites) in patients with CAVD. Compound heterozygous genotypes such as p.Phe508del/p.Arg117His and p.Phe508del/5 T are only accounted for 4% and 17% of CAVD patients^25^. Although the p.Phe508del/p.Arg117His genotype is the most common one in Caucasians. In our study, we detected only one patient with p.Phe508del variant (1.0%, 1/104), a low detection rate. 5 T variant was the most common variant identified in our cohort (54.8%, 57/104). The results suggested that there were great differences in *CFTR* variant pedigrees among different descent.

Studies has shown that in some families, even when the father, sons and brothers carried the same variant, only some of them presented with CBAVD, suggesting that a second unidentified mutations may lie in noncoding regions of the gene in CBAVD patients^26,27^, or that *CFTR* gene variant may not be the absolute influencing factor for the occurrence and development of CBAVD, and some other mechanisms and environmental factors might also be involved.

CF has been regarded as a classical autosomal recessive disorder, with no adverse health effects associated with the carrier state, and only one copy of *CFTR* variant is not enough to lead to CBAVD^4,28^. However, studies have shown that CF carriage may lead to an increased risk of CF-related diseases compared to normal control populations, but the absolute risk of CF remains low^29^. The study suggests that CF heterozygosity may be a haploid deficiency state, and the specific mechanism may be similar to thalassemia, but it needs to be studied in depth.

(TG)mTn is a multi-variant of *CFTR*, and the TG and poly T repetitions combine to affect the variable shearing of exon 10, which in turn affects the disease penetrance. In Chinese patients with CBAVD, the mutation frequency of 5 T is high, while TG12-5 T and TG13-5 T are very common in patients^30^. Previous studies have demonstrated that as the number of TG repeats increases and the number of poly T repeats decreases, the abnormal shear without exon 10 gradually increases, which is also a research direction associated with *CFTR* polymorphisms in Chinese subsequent groups of CAVD patients^31^.

In this study, 104 patients with CAVD were included. 69% (72/104) of CAVD patients carried at least one *CFTR* variant, which was basically consistent with the results of previous reports^7^. Two different variants in *CFTR* gene (compound heterozygote) were found in 60% (43/72) which might be the main cause of CAVD. Only one copy of *CFTR* variant was found in 40% (29/72). Moreover, 29.8% (31/104) of patients had no associated gene variants such as *CFTR, ADGRG2, PANK2, SLC9A3, SCNN1B* and *CA12*, suggesting that there may be unknown gene variants or pathogenic mechanism in CAVD, which need to be further studied. In addition, no hot spot *CFTR* variant was found. One case of *ADGRG2* variant was detected. We found 7 patients (6.7%, 7/104) with c.2909G > A (p.Gly970Asp) variant, which may be an important and common pathogenic variant in Chinese patients with CAVD. Possibly because of the different descent, p.Gly970Asp was detected more frequently in our study, but the sample was too small to draw a definitive conclusion and needs to be enlarged to confirm this result. Considering the economic cost, we chose whole exome sequencing. But whole exome sequencing may have some limitations. If some *CFTR* variants are located in intron, they cannot be detected. Therefore, our results showed that genetic screening based on hot spot *CFTR* variants may not be suitable for Chinese, and whole genome sequencing may be a better screening method.

There was no significant abnormality in testicular sperm in patients with obstructive azoospermia, and most CAVD couples can have biological child through ART. In our study, 81 patients with CAVD (60 patients with *CFTR* gene variants and 21 patients without *CFTR* gene variant) were treated with ICSI-ET/TESA. There were no significant differences in fertilization rate, cleavage rate, available embryo rate, implantation rate, clinical pregnancy rate and live birth rate per fresh ET whether CAVD patients had *CFTR* gene variant or not. In addition, all patients with CAVD were OA in this study, and whether the patients were CUAVD or CBAVD, we had not found the influence about *CFTR* genotypes and ART outcome.

*CFTR* gene variant is likely to be passed on to the next generation, and the offspring may have more serious CF, so an exhaustive analysis of *CFTR* gene should be performed in spouses of male patients carrying *CFTR* gene variant before ART. If they all carry variants, their male offspring may have a 50% chance of suffering from CAVD, and the risk for CF is 25% in their child^32,33^. So genetic evaluation must be carried out and preimplantation genetic testing (PGT) is recommended. However, there is a lack of huge amounts of data on the incidence rate of CF in China, and it is also lack of attention at present. In this study, 72 CAVD patients (69%) carried at least one *CFTR* variant, and then we screened their spouses, but Pathogenic or likely pathogenic *CFTR* variants were not found in spouses. After genetic evaluation, we did not choose PGT. In addition, 26 children (14 boys and 12 girls) were born. When they were born six months later, we followed them up through physical examination, scrotal color Doppler ultrasonography and clinical symptoms, and found that they were all healthy without typical CF symptoms. However, some atypical CF symptoms may occur at a later stage, so long-term follow-up is needed.

In view of the unclear pathogenic mechanism of CBAVD in some males, association with CF and the lack of heterozygous frequency data in Chinese population, our research on the mutational spectrum of *CFTR* or *ADGRG2* genes will be helpful for the diagnosis of this disease and the risk assessment of preimplantation genetics.

## Conclusion

In this study, we showed that rare *CFTR* variants may play a major role in Chinese patients with CAVD, and that variant spectrum was greatly different from other descent with no *CFTR* hot spots. The detection rate of *CFTR* gene variant was low, suggesting that there may be undiscovered gene variants or pathogenic mechanisms in CAVD which should be investigated further. Whole genome sequencing may be a more suitable genetic detection method for Chinese people. There was no significant difference in ART outcomes in CAVD patients with or without *CFTR* gene variant. However, the main limitation of this study was that we could not statistically analyze the sperm quality through TESA. In addition, due to the small sample size of our study, the results must be confirmed on a larger sample.

## Supplementary Information

Supplementary Information.

## Data Availability

The datasets generated and analysed during the current study are available in the NGDC repository (HRA001893).
